# The association between measures of subjective cognitive decline and Alzheimer’s disease plasma biomarkers in cognitively normal individuals

**DOI:** 10.1002/alz.093492

**Published:** 2025-01-09

**Authors:** Mark A Bernard, Allal Boutajangout, Ludovic Debure, Anthony Q Briggs, Alok Vedvyas, Karyn Marsh, Ricardo Osorio, Yongzhao Shao, Rebecca A Betensky, Thomas Wisniewski, Arjun V. Masurkar

**Affiliations:** ^1^ NYU Grossman School of Medicine, New York, NY USA; ^2^ NYU Alzheimer's Disease Research Center, New York, NY USA; ^3^ Center for Cognitive Neurology, NYU Grossman School of Medicine, New York, NY USA; ^4^ Center for Cognitive Neurology, New York, NY USA; ^5^ Center for Cognitive Neurology, New York University Langone Health, New York, NY USA; ^6^ Alzheimer's Disease Research Center, New York University Langone Health, New York, NY USA; ^7^ Nathan Kline Institute, Orangeburg, NY USA; ^8^ New York University Grossman School of Medicine, New York, NY USA; ^9^ NYU College of Global Public Health, New York, NY USA

## Abstract

**Background:**

Subjective cognitive decline (SCD) is thought to be a preclinical stage of Alzheimer’s disease (AD), associated with faster disease progression and with imaging and cerebrospinal fluid biomarkers. However, it is unclear whether and how the degree of SCD correlates with plasma biomarkers. To address this, we investigated the association of plasma biomarkers of AD with established validated measures of SCD.

**Method:**

The study included 122 cognitively normal older adults (age ≥60 years, 72 non‐Hispanic White, 50 Black/African American) evaluated at the NYU Alzheimer’s Disease Research Center. Participants underwent a comprehensive battery of psychometric tests according to NACC UDS 3 standards. Degree of SCD was quantified using the Brief Cognitive Rating Scale (BCRS) and Cognitive Change Index (CCI). Plasma levels of Aβ40, Aβ42, total tau, ptau‐181, neurofilament light (NfL), and glial fibrillary acidic protein (GFAP) were measured using ultra‐sensitive, single‐molecule array (SIMOA) technology. Secondarily‐calculated measures included Aβ42/Aβ40, tau/Aβ42, and ptau‐181/Aβ42 ratios. Linear regression (models unadjusted and adjusted for age) was used to evaluate the strength of association between psychometric measures and plasma biomarkers.

**Result:**

In unadjusted linear regression, both higher BCRS (β = .0420, r^2^ = .1135, F = 7.682, p = .0074) and CCI (β = .0059, r^2^ = .1636, F = 11.732, p = .0011), each indicative of a higher degree of SCD in this cognitively normal population, were found to be predictive of higher plasma tau/Aβ42 ratio. In age‐adjusted models, higher BCRS (β = .0368 p = .0087) and CCI (β = .0066, p = .0019) remained significantly predictive of higher plasma tau/Aβ42, with higher CCI additionally found to be predictive of lower plasma Aβ40 (β = ‐1.659, p = .0210). Statistically significant associations of SCD measures were not observed with Aβ42/Aβ40, ptau‐181/Aβ42, NfL, or GFAP.

**Conclusion:**

Validated measures of SCD significantly associated with tau/Aβ42 ratio and Aβ40 levels in plasma, but do not show significant association with other biomarkers. This suggests that the degree of SCD in cognitively normal individuals could potentially signify early aspects of neurodegeneration that are distinct from other pathways. This relationship warrants further study in longitudinal studies of SCD cohorts.

